# Relative progress and academic performance of graduate vs undergraduate entrants to an Australian medical school

**DOI:** 10.1186/s12909-019-1584-0

**Published:** 2019-05-22

**Authors:** Ian B. Puddey, Annette Mercer, Sandra E. Carr

**Affiliations:** 1 School of Medicine, Faculty of Health and Medical Sciences, University of Western Australia, Level 4 RPH MRF Building, Rear 50 Murray St, Perth, WA 6000 Australia; 2 Division of Health Professions Education, School of Allied Health, Faculty of Health and Medical Sciences, MB 414 University of Western Australia, 35 Stirling Hwy, Crawley, WA 6009 Australia

**Keywords:** Medical students, Graduate entry, Undergraduate entry, Academic performance, Progression

## Abstract

**Background:**

Whether graduate entrants to medical school perform better academically than undergraduate entrants remains controversial. Differences in the discipline backgrounds of graduates may, at least in part, have accounted for differences in the results of comparative studies reported to date. This study aimed to address the dual issues of whether academic performance and progression rates were different between GE and UG students and the extent to which the discipline background of GE students may underpin any differences observed.

**Methods:**

Relative academic performance as well as indicators of student progression (supplementary examinations, repeat years, leave of absence, withdrawal from the programme) were compared between graduate entrants (GE) (*N* = 410) and both school leaver entrants (SLE) (*N* = 865) and non-standard entrants (some prior tertiary education) (NSE) (*N* = 148) who combined for the final 4 yr. of a 6 yr. MBBS undergraduate programme in 8 consecutive cohorts from 2006 to 2013 in Western Australia.

**Results:**

Examination scores were generally at or very close to a distinction grade or higher across all groups. Higher mean examination scores were seen for GE versus both SLE and NSE in the first 2 years with no significant differences in the final 2 years. GE from biological science / science backgrounds (*N* = 241) or physical science backgrounds (*N* = 26) performed the same as SLE and NSE throughout the programme. GE with a health / allied health background (*N* = 91), however, performed better throughout. They also performed better when compared to their GE counterparts from a humanities (*N* = 32) or a biological science / science background. GE had increased odds of withdrawing when compared to SLE (OR 2.50, 95% CI 1.30, 4.79, *P* = 0.006), but not compared to NSE. NSE had increased odds of repeating at least one level when compared to either GE (OR 2.74, 95% CI 1.21, 6.21, *P* = 0.016) or SLE (OR 4.10, 95% CI 1.93, 8.70, *P* < 0.001). There were no differences by entry category in the odds of sitting at least one supplementary examination during the programme. There was an increase in the odds of taking at least one leave of absence in both SLE (OR 2.55, 95% CI 1.79, 3.63, *P* < 0.001) and NSE (OR 2.47, 95% CI 1.50, 4.07, P < 0.001) compared to GE.

**Conclusions:**

Better academic performance by GE compared to SLE and NSE was predominantly due to higher scores for GE with a health / allied health background. GE were also less likely to have impeded progress during the course.

**Electronic supplementary material:**

The online version of this article (10.1186/s12909-019-1584-0) contains supplementary material, which is available to authorized users.

## Background

Graduate entry into medical programmes in Australia which began in the early 1990’s, has grown substantially in the 3 decades since and is now available at 13 of the nation’s 21 medical schools. Such growth reflects what are seen as many of the proposed benefits of a graduate entry program including that such students already have a proven track record at a tertiary level with the anticipation that following a mature decision to study medicine there will be increased retention rates, increased commitment and higher motivation [1]. It has also been suggested that during their studies they will be more self-directed, challenging, demanding and questioning [2], that they will have increased goal orientation and cooperativeness [1], that they will bring superior communication skills vs undergraduate students [3] and that they will have an increased breadth of undergraduate and life experiences [4]. However, whether graduate entry programmes deliver better academic outcomes or increased retention rates, still remains controversial. There have now been 8 comparative studies of the performance of graduate entry (GE) vs undergraduate entry medical students who have been participating in programmes with identical curriculum content and assessment, but the results as summarised in Table 1 have been mixed [5–12]. Overall however, GE students tend to have performed better academically than UG students.

**Table 1:** Graduate entry vs undergraduate entry into medical school – comparative studies of academic performance and progression

Institution	Programme duration	Students	Selection Pre-requisites	Methodology	Confounding variables	Performance	Progression
University of Nottingham [5]	UG – 5 yr. GE – 4 yr	All graduates from 2007 and 2008 UG *N* = 450 GE *N* = 171 (27.5%)	No data on selection process	Comparison of final 2.5 yr. of clinical training. Exclusion from the study of those who had taken longer than the standard 4 (or 5) years to complete.	Substantially different demographic profile for GE and UG (higher age, more females). Cohort effects. Lower prior educational attainment by GE	Lower performance on 4 of 5 knowledge-based exams by GE. Similar levels of performance for GE and UG on all skills-based and attitudinal assessments	Completion rates significantly higher for GE vs UG (94% vs 90%). Similar failure rates in assessments overall
University of Birmingham [6]	UG – 5 yr. GE – 4 yr	Four successive student cohorts (2003–4 to 2006–7) UG *N* = 1386 GE *N* = 161 (10.4%)	Pre-requisites for selection - an A-level in chemistry and first degree must be in a life science	Comparison of clinical or non-clinical assessments during final 3 yr. of training	Differences in ethnicity, gender and number of international students between GE and UG	Higher average marks in the GE cohort compared to UG, mainly in the clinical examinations. Poorer performance by students from overseas, students of South Asian background or male students	Proportion of students graduating with honours was higher in the GE group.
Newcastle University, UK [7]	UG – 5 yr. GE – 4 yr	Five cohorts of students from 2001 onwards UG *N* = 1254 GE *N* = 140 (10.0%)	GE - 2.1 degree or higher in any subject, or a practising healthcare professional with equivalent academic achievement	Comparison of knowledge and critical thought assessments during final 3 yr of training Also compared GE to graduates who entered the 5 yr. programme	Background degree for GE students	GE students had consistently higher assessment scores than both UG and graduates in the 5 yr. programmeNo influence on scores of background degree	Not reported
Leicester-Warwick Medical School [8]	UG – 5 yr. GE – 4 yr	3 cohorts who completed from 2004 to 2006. UG *N* = 316 GE *N* = 285 (47.0%)	No data on selection process	Comparison at the end of the pre-clinical phase (1.5 yr. for GE, 2.5 yr. for UG). Comparison of identical 2.5 yr. clinical phase. More UG students did not consent to take part (27% vs 11% for GE) UG students with previous degrees excluded.	UG with prior degrees were excluded from the analysis. GE students had weaker prior academic performance as assessed by A-levels at secondary school.	No differences between the scores on the written examination at the end of the pre-clinical phase. UG students performed better in the intermediate clinical examination but there were no differences in the final clinical assessment. GE students performed better in the final written examinations at the end of the clinical phase.	Any student who did not complete the course to finals stage was removed from the analysis. Relative attrition rates were not compared.
Royal College of Surgeons in Ireland [9]	UG – 5-6 yr. GE – 4 yr	4 consecutive cohorts who underwent their final 2 year examinations 2008 to 2013. UG *N* = 703 GE *N* = 233 (24.9%)	GE - a level 8 honours degree with a minimum 2:1 honours classification (in any subject). Must also achieve a competitive score on either GAMSAT or MCAT.	Comparison of the final 2 yr. of the programme.	Background degree for GE students Nationality MCAT/GAMSAT entry test scores	For all years examined consistent evidence of significantly better outcomes for GE vs UG. No difference in performance between those with a science vs a non-science background.	Not reported
College of Medicine, King Saud bin Abdulaziz University of Health Sciences [10]	UG – 6 yr. GE – 4 Mth bridging course, then 4 yr	3 consecutive cohorts of male medical students 2010–2014. UG *N* = 196 GE *N* = 54 (21.6%)		Compared final 4 yr. − 2 yr. pre-clinical and 2 yr. clinical	All GE students had a health science background.	UG performed significantly better than GE on a few pre-clinical courses. No significant difference in performance between UG and GE in clinical rotations.	Not reported
University of Melbourne [11]	UG – 5-6 yr. GE – 4 yr	4 successive cohorts of medical students who entered from 2002 to 2005 UG *N* = 464 GE *N* = 240 (34.1%)	UG – performance in final secondary school assessment and the UMAT. GE - tertiary academic performance using GPA, GAMSAT score and a structured interview. All background degrees eligible.	Compared over 2 yr. during the pre-clinical component of the course. Excluded students who did not have a consecutive sequence of assessments (leave of absence, enrolment in combined degree, withdrawal from the course).	Higher proportion of GE (60%) rather than UG (52%) were female. Excluded international students.	GE performed consistently better, (but only marginally) than UG on assessments of bioscience knowledge and clinical skills. Comparison was restricted to those students who had passed each subject on their first attempt.	GE were no more likely than UG to have an interrupted sequence of subjects, to transfer course or withdraw. GE were less likely than UG to take a supplementary examination
University of Melbourne [12]	UG – 6 yr. GE – 4 yr	4 successive cohorts of medical students who completed from 2004 to 2008 UG *N* = 471 GE *N* = 242 (33.9%)	As above.	Comparison of initial and final assessments during 2 yr. of clinical training. Only students who had completed their initial and final clinical assessments in consecutive years were included.	Higher proportion of GE (58%) rather than UG (51%) were female. Excluded international students.	No differences in performance between GE and UG were seen. The performance of female students was better at both the initial and final assessments during clinical training.	All students passed their assessments on the first attempt.

The only previous comparative study from Australia was carried out at the University of Melbourne where 4 successive cohorts of medical students who entered from 2002 to 2005 were evaluated during four common semesters during the pre-clinical component of the course [11]. GE students performed consistently better, but only marginally, than UG students on assessments of bioscience knowledge and clinical skills over the 2 years. GE students were also less likely than undergraduate students to take a supplementary examination [11]. However, when a subsequent comparison was made of initial and final assessments during the clinical period of training [12] no differences in performance were seen. Therefore, whether academic performance has been assessed early in the course (pre-clinical), later in the course (clinical) or as a whole of course assessment may have contributed to the inconsistent outcomes across all studies. When pre-clinical assessments have been evaluated, GE students have either performed worse than or identically to UG students in 2 studies [5, 8] but better in four [6, 7, 10, 11], while for clinical or overall assessments throughout the course GE performance was better than UG in 3 studies [6, 7, 9] and identical in four [5, 8, 10, 12]. Academic progression as assessed by completion rates was only evaluated in 2 of the 8 studies with one showing better progression in GE vs UG students [2] and the other showing worse [8].

This lack of consistency in outcomes has several potential contributing factors. Differences in admission criteria or differences in the relative ratios of applicants to the available places may influence relative academic ability of GE vs UG cohorts as well as the ultimate sociodemographic mix in each group. Weaker prior educational attainment in graduate entry cohorts has been cited as a confounding factor [8], but for the large part, differences in prior academic abilities has not been assessed in these studies. Several sociodemographic factors may have influenced relative performance in comparisons of GE vs UG students including socioeconomic background, gender [12], age at entry to the program as well as the changing age profile of GE students over time (younger as programmes become more established) [5]. Differences between medical programmes across universities also need to be considered, especially differences between the first phase of a GE programme vs an UG medical programme (e.g. a problem based learning vs more traditional learning approach) [6, 7]. Relative numbers of GE vs UG students have varied considerably across studies to date with generally much larger sample sizes in the UG cohorts (Table 1). The relative proportion of students in UG programmes who enter with either previous tertiary experience or a previous degree has largely not been considered or such students have been excluded from the analysis [8]. Exclusion of students with interrupted progress during the course [5], exclusion of students because they did not have a consecutive sequence of assessments because of leave of absence, enrolment in combined degrees or withdrawal from the course [11] or exclusion of students who did not pass any assessment at first attempt [11] may all have increased rather than reduced bias in any comparisons.

Finally, the relative proportion of GE students from non-science backgrounds as well as the proportion of students from health-related backgrounds may have dictated many of the differences seen. In the 2 studies where discipline background of GE students was considered no differences in student performance were identified [7, 9]. At the University of Western Australia we have previously reported that amongst our GE students those with health or allied health backgrounds perform better throughout the course while those from humanities backgrounds have weaker performance, especially earlier in the course [13]. In the current study, therefore, we aimed to address the dual issues of whether academic performance and progression rates were different between GE and UG students and the extent to which the discipline background of GE students may underpin any differences observed.

## Methods

### Study participants

Eight consecutive cohorts of school leaver entrants (SLE), non-standard entrants (NSE) (at least 1 year of prior tertiary education) and graduate entrants (GE) were studied. The SLE students (*N* = 924) and NSE students (*N* = 162) entered the first year of their 6 year programme between 2004 and 2011 while GE students (*N* = 428) entered a 6 month bridging course between 2005 and 2012. From 2006 to 2013 the 8 cohorts of GE, SLE and NSE students joined levels 3–6 of the undergraduate programme, undergoing identical training and assessment thereafter.

Both the first 2 years of the 6-yr undergraduate programme and the 6 month bridging course utilised a problem-based learning approach. International students and indigenous students who entered by alternative pathways, predominantly into the 6-year programme rather than the GE programme, were excluded from the analysis. Students who entered by other quarantined entry pathways (for rural students or socio-educationally disadvantaged students) were well represented across the 3 study groups and therefore included in the final analysis.

### Setting

The SLE students were selected into the course on the basis of prior academic performance (the Australian Tertiary Admissions Rank – ATAR, 0–99.95), score on an aptitude test (the Undergraduate Medical Admissions Test – UMAT) [14] and a score out of a possible 42 from a highly structured 2-person panel interview. Embedded in the interview score were a communication skills score (out of 6) and a motivation / commitment score (also out of 6). For NSE students prior academic performance was assessed from the grade point average (GPA) achieved during their previous tertiary studies, and they also completed the UMAT and interview. For GE students, GPA and interview score were used together with a score from another aptitude test – the Graduate Australian Medical Students Admissions Test – GAMSAT [15].

### Outcomes

A comparative analysis was made of indicators of student progression over levels 3–6 which included taking at least one supplementary (repeat) examination, repeating one or more levels of the course, taking a leave of absence or withdrawal from the programme. Withdrawal was further classified as academic (where a student either was expelled from the programme or elected not to continue following failure in one or more units) or non-academic.

All students who had completed the programme by 2017 were included in the analysis of relative academic performance. The outcome variable of academic performance was compared utilising the weighted average mark (WAM) calculated for each of levels 3 to 6 of the course (with each unit weighted according to its relative contribution to the yearly mark). For those who passed a supplementary examination after an initial unit fail, the yearly WAM was calculated from an attributed mark of 50% for that unit. For those who failed a unit and then repeated a year, the yearly WAM was calculated from the mark obtained at the first assessment for that unit. Secondary outcome variables included the percentage mark given for a range of individual units which were either ‘knowledge’-based or ‘clinically’ based [16]. For the former, the curriculum was delivered mainly in didactic fashion in lectures and laboratory sessions; and assessment was predominantly of factual knowledge. For the latter, the curriculum was delivered through a combination of problem-based learning tutorials, case-based tutorials or clinical teaching; and assessment was either through a multidisciplinary observed structured clinical examination or a composite assessment of clinical performance during a clinical clerkship. The level 3 ‘clinical’ unit also had a substantial knowledge based approach in its teaching and assessment.

Both student progress and academic performance were also compared after a further breakdown of the GE students into one of 4 disciplinary backgrounds (modified from Craig et al. [17]); Biological sciences/science – single or double major in human biological sciences or non-human biological sciences; Health/allied health – pharmacy, physiotherapy, nursing, occupational therapy, exercise and health sciences, radiography, nutrition and dietetics, dentistry, veterinary medicine and other health-related professions; Humanities – arts, commerce, business, law, social science; and the Physical sciences – physics, mathematics, chemistry, engineering, information and communication technology. Psychology was classified with the Humanities if students graduated with a Bachelor of Arts but as Biological Sciences/science if they graduated with either a Bachelor of Science or a double degree in both Arts and Science.

### Statistical analysis

Data were analysed in SPSS for Windows version 24.0.0.2. Summary statistics at entry were compared across NSE, SLE and GE students by either one-way ANOVA or unpaired t-test for continuous variables or Pearson’s Chi Square for categorical variables. The distribution of each data set was evaluated using Shapiro-Wilks test. Significance levels were reported at 0.05, 0.01 and 0.001 and a Bonferroni post hoc correction was applied. Relative progression of the 3 student groups was assessed by logistic regression models with dummy dependent variables (0,1) constructed for each endpoint (i.e. taking at least one supplementary examination, repeating one or more levels, taking a leave of absence, academic and non-academic withdrawal from the programme) and entry category, gender and mode of entry (quarantined / non-quarantined pathway) as predictor variables. Relative academic performance in levels 3–6 of the course were assessed by general linear modelling (GLM) ANOVA models which included gender, a gender by entry category interaction term, mode of entry (quarantined / non-quarantined), year each level was completed and entry category (NSE, SLE and GE) as fixed factors. Goodness of fit was assessed using the Lack of Fit test for Univariate GLM.

## Results

### Baseline characteristics

Baseline characteristics for SLE, NSE and GE students are outlined in Table 2. GE were older than SLE (*P* < 0.001) and older than NSE (*P* < 0.001). The proportion of males vs females was similar across all 3 groups. NSE were represented by a higher proportion of students who had entered via quarantined entry pathways for students of rural or socio-educationally disadvantaged backgrounds. For those students for whom data was available on high school academic achievement at entry (ATAR), NSE and GE students did not perform as well as SLE. Data for academic performance during previous tertiary studies (GPA) revealed no significant difference between NSE and GE students. SLE students scored lower overall interview scores and lower scores on motivation / commitment (but not communication) relative to the GE students.

**Table 2:** Baseline characteristics of non-standard entry, school leaver entry and graduate entry students

	Non-standard entry (*N* = 148)	School Leaver entry (*N* = 865)	Graduate entry (*N* = 410)	*P* value
Age at entry (yr)	21.3 ± 0.3^** ##^	18.3 ± 0.02^**^	25.6 ± 0.3	< 0.001
Gender (M/F)	60 (40.5%)	389 (45.0%)	178 (43.4%)	0.576
Quarantined entry	50 (33.8%)	203 (23.5%)	75 (18.3%)	0.001
ATAR	95.13 ± 0.45^##^ (*N* = 122)	98.80 ± 0.04 (N = 865)	94.82 ± 0.37^##^ (*N* = 169)	< 0.001
GPA at entry	6.33 ± 0.04		6.39 ± 0.02	0.260
Interview score	26.5 ± 0.4	26.5 ± 0.2^*^	27.3 ± 0.3	0.045
Communication skills score	4.0 ± 0.1	4.2 ± 0.04	4.2 ± 0.06	0.258
Motivation / Commitment score	3.8 ± 0.09	3.7 ± 0.04 ^**^	4.0 ± 0.05	< 0.001

*P* values are from One-way ANOVA for continuous data and Pearson chi-squared tests for categorical data. Post hoc comparisons from One-way ANOVA (Bonferroni correction) - ^*^P < 0.05, ^**^P < 0.001 compared to graduate entry, ^##^*P* < 0.001 compared to school leaver entry

Twenty of the 148 NSE students had completed a prior tertiary degree while the remainder entered after completing level 1 or level 2 of a tertiary degree. In 45 NSE students the discipline backgrounds in relation to their previously completed degree or prior incomplete degree programme was not on record. For the remainder, the breakdown was Biological science / Science (*N* = 69, 67.0%), Health / Allied health (*N* = 15, 14.5%), Humanities / Commerce/ Business / Law *N* = 12 (11.7%) and Physical sciences (*N* = 7, 6.8%).

The large majority of GE students came from Biological science / Science backgrounds (*N* = 253, 61.7%), with 23.2% (*N* = 95) from Health / Allied health, 8.5% (*N* = 35) from Humanities / Commerce/ Business / Law and 6.6% (*N* = 27) from Physical sciences. This breakdown was no different than that seen for those NSE students where discipline background was known (Pearson Chi square = 4.09, *P* = 0.25). Baseline characteristics for GE students broken down by background discipline are outlined in Table 3. Health /Allied Health students and Humanities students were older than Biological science / Science students (*P* = 0.01 and *P* < 0.001, respectively). Health /Allied health students were younger than Humanities students (*P* = 0.046). Males were relatively underrepresented in those with Biological science /Science backgrounds and over-represented in those with a Physical sciences background. Students with a Biological science / Science background or a Health / Allied health background were represented by a higher proportion of students who had entered via quarantined entry pathways. For those students for whom data was available on high school academic achievement at entry (ATAR), those with a humanities background did not perform as well as those with a physical sciences background. Data for academic performance during previous tertiary studies (GPA) revealed no significant difference by background discipline.

**Table 3:** Baseline characteristics of graduate entry students broken down by background discipline

	Biological science / Science (*N* = 253)	Health / Allied Health (*N* = 95)	Humanities / Commerce / Business / Law (*N* = 35)	Physical Sciences (*N* = 27)	*P* value
Age at entry (yr)	24.6 ± 0.3	26.7 ± 0.5 ^** #^	29.3 ± 1.3 ^**^	27.0 ± 1.0	< 0.001
Gender (M/F)	97 (38.3%)	46 (48.4%)	17 (48.6%)	18 (66.7%)	0.019
Quarantined entry	36 (14.2%)	20 (21.1%)	13 (37.1%)	6 (22.2%)	0.008
ATAR	95.12 ± 0.39 (*N* = 128)	93.52 ± 0.96 (N = 24)	91.75 ± 2.60 (N = 10)	98.23 ± 0.49 ^#^ (N = 7)	0.017
GPA at entry	6.42 ± 0.02	6.33 ± 0.04	6.26 ± 0.07	6.45 ± 0.07	0.059
Interview score	27.1 ± 0.3	27.7 ± 0.5^*^	28.4 ± 1.0	25.5 ± 1.2	0.146
Communication skills score	4.1 ± 0.1	4.3 ± 0.1	4.4 ± 0.3	3.8 ± 0.3	0.166
Motivation / Commitment score	3.9 ± 0.1	4.1 ± 0.1	4.1 ± 0.2	3.7 ± 0.2	0.314

*P* values are from One-way ANOVA for continuous data and Pearson chi-squared tests for categorical data. Post hoc comparisons from One-way ANOVA (Bonferroni correction) - ^*^P < 0.05 ^**^P < 0.001 compared to Biological Science / Science, ^#^*P* < 0.05 compared to Humanities

### Programme completion levels 1–2 or GE bridging Programme

There was no significant difference for the overall completion rate for levels 1 and 2 for SLE and NSE students (1013 of 1086 students, 93.3%) when compared to the 6 month bridging programme for the GE students (410 of 428 students completed, 95.8%). However, the rate of academic withdrawal for levels 1 and 2, but not non-academic withdrawal, was higher in the SLE and NSE students (37 of 1086 students, 3.4%) vs GE students (3 of 428, 0.7%). A logistic regression model that included gender and mode of entry (quarantined / non-quarantined pathways), indicated that the odds for academic withdrawal during levels 1 and 2 for SLE and NSE students was 4.60 (95% CI 1.40, 15.04, *P* = 0.012) when compared to academic withdrawal from the bridging programme by GE students.

### Programme completion levels 3–6

For all students who commenced level 3, Levels 3 to 6 were completed by 390 of 410 GE students (95.1%), 847 of 865 (97.9%) SLE students and 142 of 148 (95.9%) NSE students (Pearson Chi square = 7.77, *P* = 0.021). All 4 levels of the programme were completed within 4 years by 355 of 390 GE (91%), 648 of 847 SLE (76.5%) and 109 of 142 NSE (76.8%) (Pearson Chi square = 37.6, *P* < 0.001).

### Relative academic performance levels 3–6

Males scored significantly lower than females at every level of the course (Table 4). Students who entered via quarantined pathways also scored significantly lower at every level of the course (Table 4). There was a significant gender by entry category interaction with more pronounced differences at most levels between male GE students and male SLE and/or NSE when compared to the differences seen for females. All final GLM ANOVA models therefore included gender, a gender by entry category interaction term, mode of entry (quarantined / non-quarantined), year each level was completed and entry category (NSE, SLE and GE) as fixed factors.

**Table 4:** Estimated weighted average mark (%) from GLM ANOVA over levels 3 to 6 according to gender and mode of entry (quarantined / non-quarantined) for students who completed all 4 levels. Each GLM model also included entry category (non-standard entry, school leaver entry and graduate entry), a gender by entry category interaction term and year each level was completed as fixed factors

	Level 3	Level 4	Level 5	Level 6
Gender				
Female (*N* = 775)	71.52 ± 0.33	71.89 ± 0.20	73.84 ± 0.21	73.18 ± 0.18
Male (*N* = 604)	69.83 ± 0.41^**^	70.38 ± 0.25^**^	69.72 ± 0.61^**^	71.19 ± 0.22^**^
Mode of entry				
Not quarantined (*N* = 1065)	72.24 ± 0.27	71.73 ± 0.17	73.35 ± 0.17	73.05 ± 0.15
Quarantined (*N* = 314)	69.10 ± 0.43^**^	70.54 ± 0.26^**^	72.18 ± 0.27^**^	71.92 ± 0.24^**^

Values are estimated mean and SEM from GLM ANOVA. Pairwise comparisons at each level, ^**^P < 0.001

**Fig. 1:**
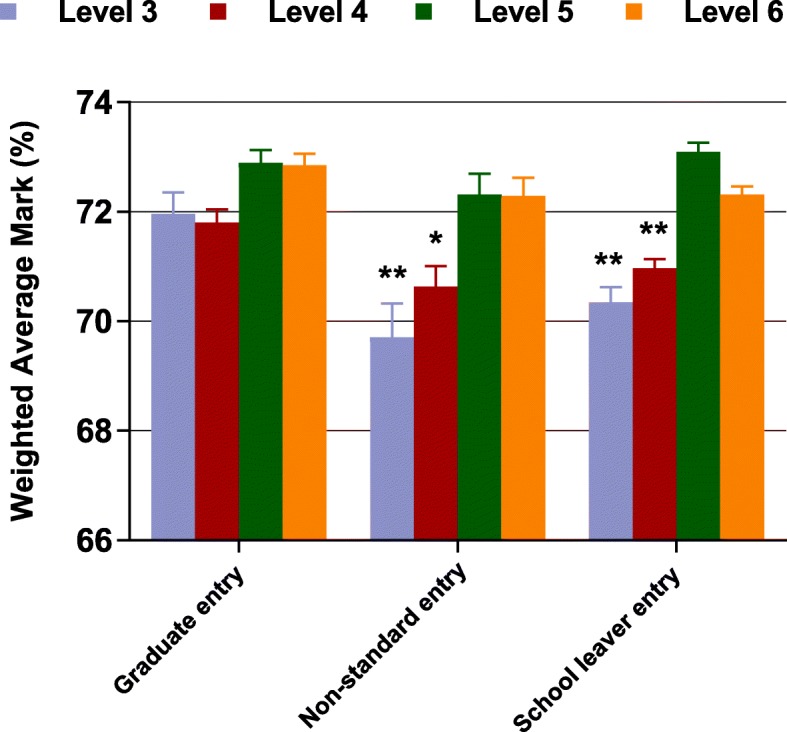
Estimated weighted average mark over levels 3 to 6 from GLM ANOVA by entry category (non-standard entry, school leaver entry and graduate entry), for students who completed all 4 levels. Each GLM model also included mode of entry (quarantined / non-quarantined), gender, a gender by entry category interaction term and year each level was completed as fixed factors. Values are estimated mean and SEM from GLM ANOVA. Post hoc comparisons with Bonferroni correction, * *P* < 0.05, ** *P* < 0.01 vs graduate

The estimated weighted average mark (%) from GLM ANOVA over levels 3 to 6 for GE, NSE and SLE students who completed all 4 levels are illustrated in Fig. 1. GE students performed significantly better than both NSE and SLE at levels 3 and 4 of the course, although the magnitude of the overall difference was small. No significant differences were evident at levels 5 and 6.

**Fig. 2:**
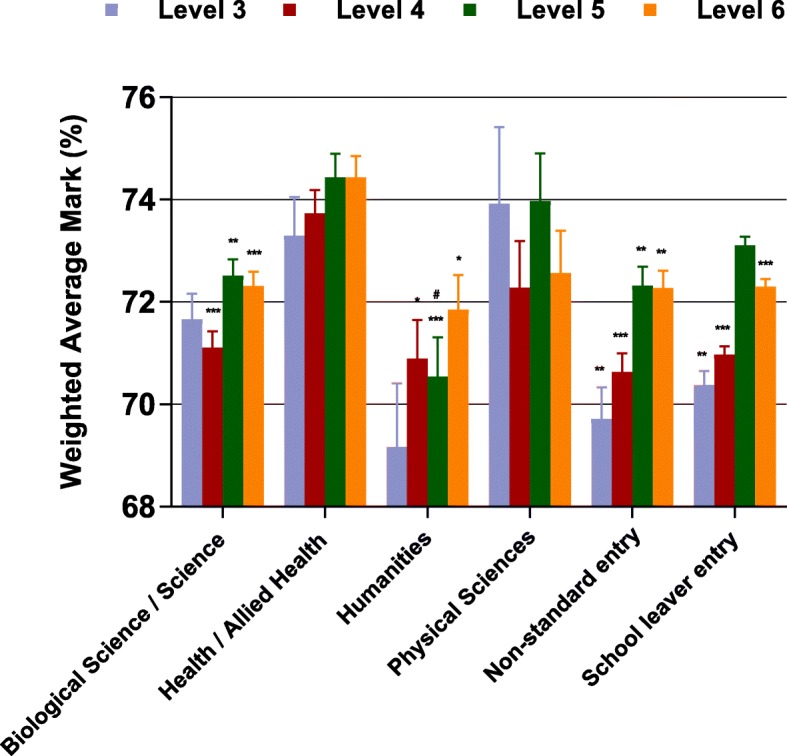
Estimated weighted average mark (%) over levels 3 to 6 from GLM ANOVA by entry category (non-standard entry, school leaver entry, graduate entry broken down by background discipline), for students who completed all 4 levels. Each GLM model also included mode of entry (quarantined / non-quarantined), gender, a gender by entry category interaction term and year each level was completed as fixed factors. Values are estimated mean and SEM from GLM ANOVA. Post hoc comparisons with Bonferroni correction, # *P* < 0.05 vs standard entry, * *P* < 0.05, ** *P* < 0.01, *** *P* < 0.001 vs health / allied health

The estimated weighted average mark (%) from GLM ANOVA over levels 3 to 6 with GE students broken down by background discipline are illustrated in Fig. 2. Students from Health / Allied health backgrounds performed significantly better than NSE at every level of the course and significantly better than SLE at levels 3, 4 and 6. They also performed significantly better than both their Biological science / Science and Humanities counterparts from levels 4 to 6 of the course. Although statistically significantly different, the overall differences seen in these generally high performing students were relatively small.

The estimated weighted average mark (%) from GLM ANOVA for knowledge-based assessments vs clinical assessments over levels 3 to 6, with GE students also broken down by background discipline, are listed in Additional file 1: Tables S1 to S8. Where there was better performance by GE versus SLE and/or NSE students, it occurred in both knowledge-based and clinical assessments at levels 3 and 4 but predominantly in clinical assessments at levels 5 and 6. The consistent trend across all levels was for this better performance to be driven by higher outcomes for those GE students with health / allied health backgrounds, with these students also generally performing better than their counterparts with humanities or biological science/science backgrounds.

### Relative Progress levels 3–6

There were no differences in the odds of overall withdrawal or withdrawal for non-academic reasons over levels 3–6 of the programme by either gender or mode of entry (quarantined / non-quarantined). The odds of withdrawal because of unsatisfactory academic progression, however, were increased for males versus females (OR 3.76, 95% CI 1.34, 10.52, *P* = 0.012). Both the odds of sitting at least one supplementary examination during the programme (OR 1.86, 95% CI 1.38, 2.51, *P* < 0.001) and the odds of sitting at least one repeat year (OR 2.67, 95% CI 1.41, 5.06, *P* = 0.003) were also increased in males versus females. There were no differences in either the odds of sitting a supplementary examination or repeating a year in students who entered via quarantined versus non-quarantined pathways. There was an increase in the odds of taking at least one leave of absence over levels 3–6 of the programme in males compared to females (OR 1.32, 95% CI 1.01, 1.72, *P* = 0.044) but no significant difference for quarantined entry compared to non-quarantined entry students.

Parameters measuring the relative progression of NSE and SLE and GE students from levels 3 to 6 are outlined in Table 5. In logistic regression models that included gender and mode of entry (quarantined / non-quarantined pathways), GE students had increased odds of withdrawing during levels 3–6 of the programme when compared to SLE (OR 2.50, 95% CI 1.30, 4.79, *P* = 0.006), but not when compared to NSE. This was predominantly due to an increase in the odds of non-academic withdrawal (OR 2.94, 95% CI 1.27, 6.80, *P* = 0.012), with no significant difference by entry category seen for withdrawal due to unsatisfactory academic progression. The main reason for non-academic withdrawal for SLE was ‘transfer to another course’ and for GE it was ‘personal reasons’.

**Table 5:** Relative progression of non-standard entry, school leaver entry and graduate entry students from levels 3 to 6

	Non-standard entry (*N* = 148)	School leaver entry (*N* = 865)	Graduate entry (*N* = 410)	*P* value
Withdrawal from the course	6 (4.1%)	18 (2.1%)	20 (4.9%)	0.020
Academic withdrawal	4 (2.7%)	8 (0.9%)	7 (1.7%)	0.159
Non-academic withdrawal	2 (1.4%)	10 (1.2%)	13 (3.2%)	0.032
At least one supplementary	27 (18.2%)	126 (14.6%)	50 (12.2%)	0.166
At least one repeat year	12 (8.1%)	19 (2.2%)	13 (3.2%)	0.001
At least one leave of absence	34 (23.0%)	201 (23.2%)	43 (10.5%)	< 0.001

*P* values are from logistic regression models with the predictor variable ‘entry category’ and school leaver entry as the reference category, in a model adjusting for mode of entry (quarantined / non-quarantined) and gender as fixed factors

NSE had increased odds of repeating at least one level during levels 3–6 of the programme when compared to either GE students (OR 2.74, 95% CI 1.21, 6.21, *P* = 0.016) or SLE (OR 4.10, 95% CI 1.93, 8.70, *P* < 0.001). There were no differences by entry category in the odds of sitting at least one supplementary examination during the programme.

There was an increase in the odds of taking at least one leave of absence over levels 3–6 of the programme in both SLE (OR 2.55, 95% CI 1.79, 3.63, *P* < 0.001) and NSE students (OR 2.47, 95% CI 1.50, 4.07, *P* < 0.001) when compared to GE students. The reasons for taking an initial leave of absence are listed in Table 6 with different patterns across the 3 student groups, travel the major reason in NSE students, travel and study the 2 major reasons for SLE students and medical and employment/ financial factors for GE students.

**Table 6:** Reason for initial leave of absence during levels 3 to 6 of the programme by mode of entry

Reason	Non-standard entry	School leaver entry	Graduate entry
Travel	15 (44.1%)	62 (30.8%)	4 (9.3%)
Study	5 (14.7%)	57 (28.4%)	2 (4.7%)
Personal	3 (8.8%)	16 (8.0%)	6 (14.0%)
Medical	4 (11.8%)	10 (5.0%)	11 (25.6%)
Employment/Financial	4 (11.8%)	41 (20.4%)	9 (20.9%)
Other	2 (5.9%)	8 (4.0%)	7 (16.3%)
Reason unknown	1 (2.9%)	7 (3.5%)	4 (9.3%)

Pearson Chi-square = 49.8, *P* < 0.001

There were no significant differences in the rate of academic or non-academic withdrawal, repeating at least 1 year or taking a leave of absence over levels 3–6 when GE students were broken down by discipline background (Table 7). Those with a Humanities background had increased odds of at least one supplementary examination over levels 3–6 compared to those from a Health / Allied health background (OR 5.42, 95% CI 1.75, 16.81, *P* = 0.003) or Biological sciences / Science background (OR 2.69, 95% CI 1.13, 6.44, *P* = 0.026).

**Table 7:** Relative progression of graduate entry students from levels 3 to 6 broken down by background discipline

	Biological science / Science (*N* = 253)	Health / Allied Health (*N* = 95)	Humanities / Commerce / Business / Law (*N* = 35)	Physical Sciences (*N* = 27)	*P* value
Withdrawal from the course	12 (4.7%)	4 (4.2%)	3 (8.6%)	1 (3.7%)	0.812
Academic withdrawal	5 (2.0%)	1 (1.1%)	1 (2.9%)	0 (0%)	0.889
Non-academic withdrawal	7 (2.8%)	3 (3.2%)	2 (5.7%)	1 (3.7%)	0.951
At least one supplementary	30 (11.9%)	6 (6.3%)	9 (25.7%)	5 (18.5%)	0.024
At least one repeat year	5 (2.0%)	3 (3.2%)	4 (11.4%)	1 (3.7%)	0.109
At least one leave of absence	25 (9.9%)	8 (8%)	7 (20.0%)	3 (11.1%)	0.205

*P* values are from logistic regression models with the predictor variable ‘background discipline’ and Biological science / Science as the reference category, in a model adjusting for mode of entry (quarantined / non-quarantined) and gender as fixed factors

## Discussion

This study has demonstrated higher mean scores in academic performance by GE versus both SLE and NSE students at levels 3 and 4 of this 6 yr. undergraduate MBBS programme, albeit by relatively small increments in what were generally high performing students. This finding of better academic performance by GE students is consistent with, and strengthens observations from the majority of previous reports [6–9, 11], but highlights the potential confounding influence of background discipline with better academic performance extending across all 4 levels of the programme when students from a Health / Allied health background were analysed separately. This also extended to higher academic scores relative to their GE peers from Biological science / Science and Humanities backgrounds.

Evidence from the United States, where medical students complete a degree programme prior to entering medical school, suggests that students with humanities backgrounds have a similar or higher standard of course performance than students with science backgrounds [18–22]. Students from health-related backgrounds, however, were not generally represented in those studies and in one, the attrition rate during the course was highest for those who had majored in the humanities and the arts [21]. Studies from the graduate entry programmes at the University of Wales [23] and Newcastle University [7] in the UK have found no relationship between prior academic background and performance in either the first 2 years or final 3 years of the course, while the relative experience in Australia has been mixed. At the University of Newcastle Medical School, Australia, there was lower academic performance of graduate entrants at first assessment in the first year in those with either an arts or nursing background and again in first year at the final assessment for those with a nursing background [24]. In a combined study of graduate entry medical students at the University of Queensland and the University of Sydney [25], academic performance as measured by second year examination results, was lower in students with a non-biological science based primary degree. These students also scored lower in one of two instruments utilised for assessing clinical reasoning. In a further study from the University of Sydney, students with science based degrees and those from health professional backgrounds obtained higher performance scores during the first 3 years of a graduate entry medical programme and had lower failure rates, but these differences were considered small and diminished over time [17].

That graduate entrants perform at least as well as, if not better than school leaver entrants into medicine [5–12], occurs despite often having lower school-leaving results [8] as well as a shorter overall period of training at medical school. This has been attributed to their learning approach, a recent review finding that more graduates than undergraduates utilise strategic/deep learning approaches rather than surface learning approaches [26]. Higher academic performance by students with health professional backgrounds has been variously attributed to familiarity with medical knowledge and prior experience in the clinical setting [17]. Our health / allied health background GE students were older than both the SLE and NSE groups as well as their biological science/ science counterparts raising the question of the additional role of maturity. Whether maturity alone can be invoked as a factor in the better performance is doubtful, however, given that our oldest group, those from a humanities non-science background, generally performed at a lower level throughout the programme. Mature-age British medical students have been shown to perform better overall than school leaver entrants during the first 2 preclinical years [27] but Australian studies have not repeated this observation. A comparison of 121 mature-age and 270 normal-age entrants who graduated from the University of Queensland Medical School between 1972 and 1987 found the whole of course grades were similar in both groups [28]. Similarly, the performance of all entrants to the University of Newcastle Medical School, Australia, from 1994 to 1997, when assessed twice during the first year [29], reported that, although older students were more likely to receive a satisfactory rather than non-satisfactory rating at the first assessment, there was no longer a difference in the final assessment.

The absolute differences in relative academic performance between the GE vs SLE and NSE students in this study were relatively small, all mean levels generally at or very close to a distinction grade or higher (WAM ≥ 70%) across the study groups at each level of the programme. However, academically high achieving students were necessarily recruited into the programme and so it is not surprising that the documented differences were small. The relative differences were larger when the Health / Allied health students were assessed against SLE and NSE students, while the relative under-performance of those from a Humanities background would have simultaneously diminished absolute differences between GE vs SLE and NSE students. The differences also need to be considered in the light of the relative progression of the GE vs SLE and NSE students prior to the commencement of Level 3. The progress status of the SLE and NSE students over levels 1–2 of the course was characterised by greater rates of withdrawal for academic reasons compared to GE students during their bridging course and so the SLE and NSE students had already been enriched by removal of more under-performing students by the commencement of Level 3. The low rates of academic withdrawal even in SLE and NSE (3.4% for level 1–2) is acknowledged and the very wide confidence intervals indicate lingering uncertainty as to whether prior programme completion rates were a confounding factor in the study outcomes.

We included in our final multivariate models the year each level (3 to 6) was sat and so those with impeded progress due to leave of absence or enrolment in a combined degree could still be included in the final analysis. There was evidence of impeded progress in the SLE and NSE students over levels 3–6 of the programme compared to GE students who were significantly more likely to complete within a 4 year timeframe. This was predominantly due to more SLE and NSE students taking a year’s leave of absence for reasons of travel or study. Given this significantly higher rate for taking leave of absence, the exclusion of students with impeded progress may have biased outcomes in some previous studies [5, 11]. When GE students took a leave of absence, it was more likely for medical or financial / employment reasons, consistent with previous indications that mature-age entrants experienced greater stress with regard to financial difficulties, family problems and balancing commitments during a medical course [28, 30]. Such factors may have also contributed to the greater rate of non-academic withdrawal by GE students, withdrawal that was predominantly ascribed to ‘personal reasons’ or ‘transfer to another course’. NSE students had greater odds of needing to repeat a year compared to both SLE and GE students. Otherwise, the overall rates of academic withdrawal were similar for SLE, NSE and GE students. Within the GE students, discipline background did not appear to make any substantial difference to student progress, although those with a Humanities background were more likely to have sat at least one supplementary examination. In the only other Australian comparative study [11] GE were no more likely than undergraduate entry students to have an interrupted sequence of subjects, to transfer course or to withdraw, while in a British study, although the percentage of both GE students and undergraduate students graduating after uninterrupted progress was high, the proportion was greater in GE students.

Students with prior degrees who have entered undergraduate programmes have been included in some analyses [7] but excluded in others [8]. The inclusion of NSE students in this study allowed a comparison between GE students and those in the undergraduate programme who had either completed a prior degree or already had some tertiary level experience and success. The GPA at entry to the course was no different from that of the GE students and there are more applicants for places as a NSE student at our medical school than either as an SLE or a GE student. Yet NSE students still did not perform as well as the GE students and, as discussed previously, were more likely than both SLE and GE to repeat a year. Given that the large majority of the NSE cohort (~ 86%) were at end of level 1 or 2 of their previous tertiary programmes before entering medical school, this argues for successful completion of a tertiary programme and / or a period of time in a professional working environment as the main basis for the better academic performance of GE students, particularly for those with Health / Allied health backgrounds.

In terms of selection criteria into medical school, prior academic performance is the pre-eminent predictor of subsequent academic performance at a tertiary or post-graduate level [31]. Academic performance at a secondary school level as measured by ATAR was the main predictor of subsequent academic performance of SLE students at our institution [16]. Likewise prior academic performance at a tertiary level was the strongest predictor of subsequent academic performance for our graduate entry students [13]. The ATAR as a marker of prior relative academic performance was only available in a subset of the GE students and they had lower mean ATAR scores than the SLE students. To the extent that this measure is potentially representative of the larger group, this lower educational attainment at secondary school level might have been expected to have resulted in lower overall academic performance by the GE vs SLE students. Similarly, no difference in GPA at entry to medical school in the GE vs NSE students might be expected to predict no difference in the academic performance of GE vs NSE students. The ultimate result, with better outcomes for the GE vs both SLE and NSE students, would argue for no substantial influence of prior academic performance at a secondary or tertiary level as an explanatory factor for the better academic performance we saw in GE vs both SLE and NSE students.

The SLE and NSE students were selected into the programme by performance in a different aptitude test (the UMAT) to the GE students (the GAMSAT). Recently it has been demonstrated in a study from 2 Australian medical schools [32] that the relative variance in academic performance during the medical programme is predicted more strongly by the GAMSAT than the UMAT. The nature of these two aptitude tests is dissimilar; the GAMSAT is an applied cognitive reasoning test, assuming a fundamental knowledge base in relation to chemistry, biology, and physics, together with appropriate literacy and numeracy [15]. In contrast, UMAT is curriculum-free and is intended to provide evidence of reasoning ability additional to academic achievement as measured by the ATAR [14]. Consequently, selection of GE students by GAMSAT rather than UMAT may have been a contributor to the subsequent difference seen in academic performance of GE vs SLE and NSE students. In other comparative studies, however, a potential influence of either the GAMSAT or the Medical College Admission Test (MCAT) in dictating better academic outcomes for GE students has been dismissed either because of relatively weak (although still significant) correlations with composite performance scores during the course [9] or absent correlation with measures of tertiary academic performance [11].

With respect to the other selection criteria used for entry into our programme, the score from a standardised interview, GE students also performed better than SLE students. Of interest, the score obtained within the interview for communication skills was no different between GE vs SLE and NSE students, while the score given for questions on motivation and commitment to a career in medicine was significantly higher in GE vs SLE students, but not vs NSE students. Higher motivation and increased commitment have been previously invoked for GE students [1] and may have been a contributor to their better academic performance in the present study, while evidence for better communication skills as a potential factor [3] was lacking.

Gender may have been a confounding factor in previous studies, females generally performing better than males [6, 12], and females constituting a lower proportion of the GE versus undergraduate cohorts in one study [5] and a greater proportion in others [6, 11, 12]. In our study, females also outperformed males at every level of the course and males had higher academic withdrawal rates and greater odds of sitting a supplementary examination or repeating a year. The relative proportion of females, was similar however, across the 3 study groups. Of interest there was a gender by entry category interaction term with more pronounced differences in academic performance at most levels between GE students and SLE and/or NSE students in males compared to the differences seen for females. There was also a gender imbalance when the GE students were broken down by discipline background with males relatively underrepresented in those with Biological science /Science backgrounds and over-represented in those from a Physical sciences background. The differences in academic performance reported for either entry category or discipline background persisted after inclusion of both gender and a gender by entry category interaction term in our models.

In the long term, the more important outcome is whether graduate entry students perform better than undergraduate entry students once they are in the medical workforce and this should be the focus of any future comparative studies. In the UK, performance at medical school predicted subsequent performance in the Membership of the Royal Colleges of Physicians (UK) examination as well as being on the General Medical Council Specialist Register [31]. When we have previously assessed our graduates for independent predictors of their performance as junior doctors, the strongest has been a composite score of academic performance throughout medical school [33]. This score best predicted overall junior doctor performance, together with a score for clinical management and a score for communication skills, findings which might predict better performance by GE students as junior doctors. In the only previous report from Australia to have evaluated practice outcomes of students who entered medical school directly from high school against those who entered with some prior tertiary level education [30], no differences were seen in research outcomes, career positions held by clinicians, choice of family practice or other specialty, and practice location (rural or urban). There were also no differences between the 2 groups when subsequently compared in relation to their mean supervisor ratings at the end of their initial postgraduate intern year [34].

### Study strengths and limitations

In this report we have summarised the strengths and weaknesses of previous studies that have attempted to determine whether the academic performance and relative progression of graduate entry students is any different from undergraduate entrants. We have addressed previous methodological issues by careful ascertainment of discipline background of GE students, electing to include undergraduate students with prior academic experience as one of the comparison groups, allowing for cohort effects and analysing both the academic performance of those who have completed the programme as well as the relative progress of those who have withdrawn for either academic or non-academic reasons. Those with impeded progress were included in the final analysis and issues related to gender or quarantined entry into the course were controlled for in our final multivariate models. The numbers of non-standard and graduate entry students were smaller, there were relatively small numbers of students from humanities and physical sciences backgrounds in this study and all groups tended to have low attrition rates, potentially limiting the analysis particularly the regression.

Potential confounding from the factors utilised for initial medical student selection has been discussed and raises the possibility that the differences we have seen may have been specific to the selection processes utilised at our institution, possibly limiting the generalisability of the results to other Australian or international medical schools.

## Conclusion

The results of this study are consistent with the majority of prior reports and offer additional support to the notion that graduate entry medical students perform better academically in a medical programme than their undergraduate entry counterparts, especially in the earlier levels of the course. Better performance throughout the programme was seen particularly for those graduates with a health / allied health background. The results also indicated that graduate entry students were less likely to have impeded progress during a medical programme. Viewing these results in context, however, needs to acknowledge that these were generally all high performing students with high mean scores and high completion rates.

## Additional file

Additional file 1: **Table S1.** Estimated unit marks (%) over level 3 from GLM ANOVA by entry category (non-standard entry, school leaver entry and graduate entry), for students who completed all 4 levels. **Table S2.** Estimated unit marks (%) over level 3 from GLM ANOVA by entry category (non-standard entry, school leaver entry and graduate entry broken down by background discipline), for students who completed all 4 levels. **Table S3.** Estimated unit marks (%) over level 4 from GLM ANOVA by entry category (non-standard entry, school leaver entry and graduate entry), for students who completed all 4 levels. **Table S4.** Estimated unit marks (%) over level 4 from GLM ANOVA by entry category (non-standard entry, school leaver entry and graduate entry broken down by background discipline), for students who completed all 4 levels. **Table S5.** Estimated unit marks (%) over level 5 from GLM ANOVA by entry category (non-standard entry, school leaver entry and graduate entry), for students who completed all 4 levels. **Table S6.** Estimated unit marks (%) over level 5 from GLM ANOVA by entry category (non-standard entry, school leaver entry and graduate entry broken down by background discipline), for students who completed all 4 levels. **Table S7.** Estimated unit marks (%) over level 6 from GLM ANOVA by entry category (non-standard entry, school leaver entry and graduate entry), for students who completed all 4 levels. **Table S8.** Estimated unit marks (%) over level 6 from GLM ANOVA by entry category (non-standard entry, school leaver entry and graduate entry broken down by background discipline), for students who completed all 4 levels. (DOCX 25 kb)

## Abbreviations

ANOVA: Analysis of variance
ATAR: Australian Tertiary Admissions Rank
CI: Confidence interval
GAMSAT: Graduate Australian Medical Students Admissions Test
GE: Graduate entrants
GLM: General linear modelling
GPA: Grade point average
MBBS: Bachelor of Medicine and Bachelor of Surgery
NSE: Non-standard entrants
OR: Odds ratio
SLE: School leaver entrants
SPSS: Statistical Package for the Social Sciences
UMAT: Undergraduate Medical Admissions Test
WAM: Weighted average mark
