# Supplementary material for: Relative progress and academic performance of graduate vs undergraduate entrants to an Australian medical school
Source: BMC Med Educ. 2019 May 22;19:159. doi: 10.1186/s12909-019-1584-0 (PMC6530006; doi:10.1186/s12909-019-1584-0)
Supplement: Supplementary file 1 — Table S1. Estimated unit marks (%) over level 3 from GLM ANOVA by entry category (non-standard entry, school leaver entry and graduate entry), for students who completed all 4 levels. Table S2. Estimated unit marks (%) over level 3 from GLM ANOVA by entry category (non-standard entry, school leaver entry and graduate entry broken down by background discipline), for students who completed all 4 levels. Table S3. Estimated unit marks (%) over level 4 from GLM ANOVA by entry category (non-standard entry, school leaver entry and graduate entry), for students who completed all 4 levels. Table S4. Estimated unit marks (%) over level 4 from GLM ANOVA by entry category (non-standard entry, school leaver entry and graduate entry broken down by background discipline), for students who completed all 4 levels. Table S5. Estimated unit marks (%) over level 5 from GLM ANOVA by entry category (non-standard entry, school leaver entry and graduate entry), for students who completed all 4 levels. Table S6. Estimated unit marks (%) over level 5 from GLM ANOVA by entry category (non-standard entry, school leaver entry and graduate entry broken down by background discipline), for students who completed all 4 levels. Table S7. Estimated unit marks (%) over level 6 from GLM ANOVA by entry category (non-standard entry, school leaver entry and graduate entry), for students who completed all 4 levels. Table S8. Estimated unit marks (%) over level 6 from GLM ANOVA by entry category (non-standard entry, school leaver entry and graduate entry broken down by background discipline), for students who completed all 4 levels. (DOCX 25 kb) [file 12909_2019_1584_MOESM1_ESM.docx]

**Additional Tables S1 to S8**

Every GLM model in each of these additional tables also includes mode of entry (quarantined / non-quarantined), gender, a gender by entry category interaction term and the year each level was completed as fixed factors.

**Table S1.** Estimated unit marks (%) over level 3 from GLM ANOVA by entry category (non-standard entry, school leaver entry and graduate entry), for students who completed all 4 levels.

| **Knowledge-based assessments** | | | **Clinical and knowledge-based assessments** |  |
| --- | --- | --- | --- | --- |
| **Entry category** | **Infectious Diseases** | **Pathology** | **Medical Pharmacology** | **Fundamentals of Clinical Practice** |
| **Non-standard entry** (N=142) | 71.12 ± 0.82^***^ | 65.08 ± 0.71 | 68.01 ± 0.80^**^ | 73.04 ± 0.51^**^ |
| **School leaver entry** (N=847) | 71.79 ± 0.36^***^ | 65.86 ± 0.31 | 68.90 ± 0.35^**^ | 73.37 ± 0.23^***^ |
| **Graduate entry** (N=390) | 74.87 ± 0.53 | 66.21 ± 0.45 | 70.87 ± 0.52 | 74.95 ± 0.33 |

Values are estimated mean and SEM from GLM ANOVA. Post hoc comparisons with Bonferroni correction, ** P<0.01, *** P<0.001 vs graduate

**Table S2.** Estimated unit marks (%) over level 3 from GLM ANOVA by entry category (non-standard entry, school leaver entry and graduate entry broken down by background discipline), for students who completed all 4 levels.

| **Knowledge-based assessments** | | | **Clinical and knowledge-based assessments** |  |
| --- | --- | --- | --- | --- |
| **Entry category** | **Infectious Diseases** | **Pathology** | **Medical Pharmacology** | **Fundamentals of Clinical Practice** |
| **Non-standard entry** (N=142) | 71.18 ± 0.82^* #^ | 65.11 ± 0.70 | 68.02 ± 0.80 | 73.04 ± 0.51^***^ |
| **School leaver entry** (N=847) | 71.82 ± 0.36^** ##^ | 65.89 ± 0.31 | 68.94 ± 0.35 ^§^ | 73.37 ± 0.23^***^ |
| **Graduate entry**  Biological science / Science (N=241)  Health / Allied health (N=91)  Humanities (N=32)  Physical sciences (N=26) | 74.68 ± 0.67  76.05 ± 1.01  71.55 ± 1.66  77.16 ± 1.99 | 65.91 ± 0.58  67.70 ± 0.86  62.65 ± 1.43^*^  69.15 ± 1.71 | 70.82 ± 0.65  71.66 ± 1.02  67.17 ± 1.61  74.48 ± 1.94 | 74.44 ± 0.42  76.44 ± 0.62  74.01 ± 1.03  75.35 ± 1.24 |

Values are estimated mean and SEM from GLM ANOVA. Post hoc comparisons with Bonferroni correction, § P<0.05 vs Physical sciences, # P<0.05, ## P<0.01 vs Biological science / Science, * P<0.05, ** P<0.01, *** P<0.001 vs health / allied health.

**Table S3.** Estimated unit marks (%) over level 4 from GLM ANOVA by entry category (non-standard entry, school leaver entry and graduate entry), for students who completed all 4 levels.

| **Knowledge-based assessments** | | | | **Clinical assessments** | | | |  |
| --- | --- | --- | --- | --- | --- | --- | --- | --- |
| **Entry category** | **Infectious Diseases** | **Pathology** | **Medical Pharmacology** | **Science and Practice of Medicine** | **Clinical Skills OSCE** | **Medical Clerkship** | **Surgery Clerkship** | **Psychiatry Clerkship** |
| **Non-standard entry** (N=142) | 72.07 ± 0.70 | 69.00 ± 0.72^*^ | 67.98 ± 0.67 | 71.94 ± 0.53 | 69.16 ± 0.69 | 67.59 ± 0.47 | 69.71 ± 0.62^*^ | 67.34 ± 0.66 |
| **School leaver entry** (N=847) | 72.54 ± 0.31^*^ | 70.01 ± 0.32 | 67.63 ± 0.30^**^ | 72.01 ± 0.24 | 69.73 ± 0.31 | 67.48 ± 0.21^**^ | 69.94 ± 0.28^**^ | 67.42 ± 0.29^**^ |
| **Graduate entry** (N=390) | 73.85 ± 0.45 | 71.14 ± 0.47 | 69.31 ± 0.43 | 72.21 ± 0.34 | 70.27 ± 0.44 | 68.51 ± 0.30 | 71.48 ± 0.40 | 68.97 ± 0.42 |

Values are estimated mean and SEM from GLM ANOVA. Post hoc comparisons with Bonferroni correction, * P<0.05, ** P<0.01 vs graduate

**Table S4.** Estimated weighted average mark (%) over level 4 from GLM ANOVA by entry category (non-standard entry, school leaver entry and graduate entry broken down by background discipline), for students who completed all 4 levels.

| **Knowledge-based assessments** | | | | **Clinical assessments** | | | |  |
| --- | --- | --- | --- | --- | --- | --- | --- | --- |
| **Entry category** | **Infectious Diseases** | **Pathology** | **Medical Pharmacology** | **Science and Practice of Medicine** | **Clinical Skills OSCE** | **Medical Clerkship** | **Surgery Clerkship** | **Psychiatry Clerkship** |
| **Non-standard entry** (N=142) | 72.07 ± 0.70^**^ | 69.00 ± 0.72^*^ | 67.97 ± 0.67^*^ | 71.94 ± 0.53 | 69.14 ± 0.68^**^ | 67.57 ± 0.47^***^ | 69.71 ± 0.62 | 67.33 ± 0.65* |
| **School leaver entry** (N=847) | 72.57 ± 0.31^**^ | 70.05 ± 0.32 | 67.63 ± 0.30^**^ | 72.01 ± 0.23^**^ | 69.72 ± 0.31^***^ | 67.48 ± 0.21^***^ | 69.94 ± 0.28 | 67.42 ± 0.29^**^ |
| **Graduate entry**  Biological science (N=241)  Health / Allied health (N=91)  Humanities (N=32)  Physical sciences (N=26) | 73.39 ± 0.58  75.85 ± 0.86  71.27 ± 1.42  74.89 ± 1.71 | 70.93 ± 0.59  72.55 ± 0.88  67.72 ± 1.46  72.96 ± 1.75 | 68.49 ± 0.55  71.16 ± 0.82  68.27 ± 1.36  71.50 ± 1.64 | 71.55 ± 0.43^**^  74.32 ± 0.64  71.27 ± 1.06  72.53 ± 1.28 | 69.35 ± 0.56^***^  73.43 ± 0.84  69.22 ± 1.39  68.01 ± 1.67 | 67.81 ± 0.39^***^  70.64 ± 0.57  67.08 ± 0.95^*^  68.60 ± 1.14 | 71.16 ± 0.51  71.88 ± 0.76  70.89 ± 1.26  73.47 ± 1.51 | 68.23 ± 0.54  70.83 ± 0.80  67.58 ± 1.33  69.89 ± 1.60 |

Values are estimated mean and SEM from GLM ANOVA. Post hoc comparisons with Bonferroni correction, * P<0.05, ** P<0.01, *** P<0.001 vs health / allied health.

**Table S5.** Estimated unit marks (%) over level 5 from GLM ANOVA by entry category (non-standard entry, school leaver entry and graduate entry), for students who completed all 4 levels.

| **Knowledge-based assessment** | **Clinical assessments** | | | | |  |
| --- | --- | --- | --- | --- | --- | --- |
| **Entry category** | **Science and Practice of Medicine** | **Clinical Skills OSCE** | **Medical Clerkship** | **Obstetrics and Gynaecology Clerkship** | **Paediatric Clerkship** | **General Practice Clerkship** |
| **Non-standard entry** (N=142) | 71.54 ± 0.51 | 67.56 ± 0.62 | 74.58 ± 0.49 | 73.14 ± 0.50 | 72.27 ± 0.46 | 76.15 ± 0.51^**^ |
| **School leaver entry** (N=847) | 72.39 ± 0.23 | 68.73 ± 0.28 | 74.75 ± 0.22 | 73.86 ± 0.23 | 72.39 ± 0.21 | 76.73 ± 0.23^**^ |
| **Graduate entry** (N=390) | 71.78 ± 0.33 | 68.03 ± 0.40 | 74.89 ± 0.32 | 73.00 ± 0.33 | 72.63 ± 0.30 | 77.93 ± 0.33 |

Values are estimated mean and SEM from GLM ANOVA. Post hoc comparisons with Bonferroni correction, * P<0.05, ** P<0.01 vs graduate, OSCE – Observed Structured Clinical Examination

**Table S6.** Estimated weighted average mark (%) over level 5 from GLM ANOVA by entry category (non-standard entry, school leaver entry and graduate entry broken down by background discipline), for students who completed all 4 levels.

| **Knowledge-based assessment** | **Clinical assessments** | | | | |  |
| --- | --- | --- | --- | --- | --- | --- |
| **Entry category** | **Science and Practice of Medicine** | **Clinical Skills OSCE** | **Medical Clerkship** | **Obstetrics and Gynaecology Clerkship** | **Paediatric Clerkship** | **General Practice Clerkship** |
| **Non-standard entry** (N=142) | 71.55 ± 0.51 | 67.56 ± 0.62 | 74.57 ± 0.49 | 73.14 ± 0.50 | 72.27 ± 0.46 | 76.15 ± 0.51^***^ |
| **School leaver entry** (N=847) | 72.42 ± 0.23 | 68.76 ± 0.28 | 74.74 ± 0.22^*^ | 73.88 ± 0.23 | 72.40 ± 0.21^*^ | 76.74 ± 0.23^***^ |
| **Graduate entry**  Biological science (N=241)  Health / Allied health (N=91)  Humanities (N=32)  Physical sciences (N=26) | 71.66 ± 0.42  72.84 ± 0.63  69.44 ± 1.04  72.51 ± 1.25 | 67.79 ± 0.51  70.17 ± 0.76  63.91 ± 1.26^*** ##^  68.10 ± 1.52 | 74.05 ± 0.40^**^  76.56 ± 0.60  74.28 ± 0.99^*^  76.76 ± 1.19 | 72.84 ± 0.42  74.13 ± 0.62  70.55 ± 1.02^* #^  73.93 ± 1.23 | 72.23 ± 0.38^*^  74.22 ± 0.57  70.43 ± 0.94^**^  73.96 ± 1.13 | 77.48 ± 0.42^*^  79.75 ± 0.62  75.83 ± 1.03^*^  79.08 ± 1.24 |

Values are estimated mean and SEM from GLM ANOVA. Post hoc comparisons with Bonferroni correction, # P<0.05, ## P<0.01 vs standard entry, * P<0.05, ** P<0.01, *** P<0.001 vs health / allied health.

**Table S7.** Estimated unit marks (%) over level 6 from GLM ANOVA by entry category (non-standard entry, school leaver entry and graduate entry), for students who completed all 4 levels.

| **Knowledge-based assessment** | **Clinical assessments** | | | | |  |
| --- | --- | --- | --- | --- | --- | --- |
| **Entry category** | **Science and Practice of Medicine** | **Medical Clerkship** | **Surgery Clerkship** | **Psychiatry Clerkship** | **Emergency Medicine Clerkship** | **Rural General Practice Clerkship** |
| **Non-standard entry** (N=142) | 69.90 ± 0.36 | 70.92 ± 0.51 | 70.11 ± 0.60 | 75.61 ± 0.60 | 73.82 ± 0.57 | 76.54 ± 0.60^*^ |
| **School leaver entry** (N=847) | 70.05 ± 0.16 | 71.02 ± 0.23 | 69.53 ± 0.27 | 75.69 ± 0.27 | 73.66 ± 0.26 | 77.43 ± 0.27 |
| **Graduate entry** (N=390) | 70.33 ± 0.24 | 71.47 ± 0.33 | 70.08 ± 0.39 | 76.22 ± 0.39 | 74.18 ± 0.37 | 78.41 ± 0.39 |

Values are estimated mean and SEM from GLM ANOVA. Post hoc comparisons with Bonferroni correction, * P<0.05 vs graduate

**Table S8.** Estimated weighted average mark (%) over level 6 from GLM ANOVA by entry category (non-standard entry, school leaver entry and graduate entry broken down by background discipline), for students who completed all 4 levels.

| **Knowledge-based assessment** | **Clinical assessments** | | | | |  |
| --- | --- | --- | --- | --- | --- | --- |
| **Entry category** | **Science and Practice of Medicine** | **Medical Clerkship** | **Surgery Clerkship** | **Psychiatry Clerkship** | **Emergency Medicine Clerkship** | **Rural General Practice Clerkship** |
| **Non-standard entry** (N=142) | 69.90 ± 0.36^*^ | 70.91 ± 0.51^**^ | 70.10 ± 0.60 | 75.59 ± 0.60 | 73.81 ± 0.56^*^ | 76.53 ± 0.59^***^ |
| **School leaver entry** (N=847) | 70.06 ± 0.16^**^ | 71.00 ± 0.23^***^ | 69.51 ± 0.27 | 75.67 ± 0.27 | 73.65 ± 0.25^***^ | 77.42 ± 0.27^***^ |
| **Graduate entry**  Biological science (N=241)  Health / Allied health (N=91)  Humanities (N=32)  Physical sciences (N=26) | 69.88 ± 0.30^**^  71.73 ± 0.45  69.22 ± 0.74^*^  70.03 ± 0.89 | 70.51 ± 0.42^***^  73.97 ± 0.63  69.55 ± 1.04^**^  71.58 ± 1.25 | 70.02 ± 0.50  70.09 ± 0.74  70.45 ± 1.22  69.91 ± 1.47 | 75.91 ± 0.50  77.51 ± 0.74  76.57 ± 1.22  74.71 ± 1.47 | 73.49 ± 0.47^**^  76.70 ± 0.69  72.33 ± 1.15^*^  73.89 ± 1.38 | 77.55 ± 0.49^**^  80.72 ± 0.73  76.37 ± 1.21^*^  78.61 ± 1.46 |

Values are estimated mean and SEM from GLM ANOVA. Post hoc comparisons with Bonferroni correction, * P<0.05, ** P<0.01, *** P<0.001 vs health / allied health.
